# Investigating Associations Between Inflammatory Biomarkers, Gray Matter, Neurofilament Light and Cognitive Performance in Healthy Older Adults

**DOI:** 10.3389/fnagi.2021.719553

**Published:** 2021-09-03

**Authors:** Hollis C. Karoly, Carillon J. Skrzynski, Erin Moe, Angela D. Bryan, Kent E. Hutchison

**Affiliations:** ^1^Institute for Cognitive Science, University of Colorado Boulder, Boulder, CO, United States; ^2^Department of Psychology, Colorado State University, Fort Collins, CO, United States; ^3^Department of Psychology and Neuroscience, University of Colorado Boulder, Boulder, CO, United States; ^4^Department of Psychiatry, University of Colorado Anschutz Medical Campus, Aurora, CO, United States

**Keywords:** aging, gray matter, neurofilament light (NfL), IL-6, cognitive function

## Abstract

**Background**: Exploring biological variables that may serve as indicators of the development and progression of cognitive decline is currently a high-priority research area. Recent studies have demonstrated that during normal aging, individuals experience increased inflammation throughout the brain and body, which may be linked to cognitive impairment and reduced gray matter volume in the brain. Neurofilament light polypeptide (NfL), which is released into the circulation following neuronal damage, has been proposed as a biomarker for neurodegenerative diseases, and may also have utility in the context of normal aging. The present study tested associations between age, peripheral levels of the pro-inflammatory cytokine IL-6, peripheral NfL, brain volume, and cognitive performance in a sample of healthy adults over 60 years old.

**Methods**: Of the 273 individuals who participated in this study, 173 had useable neuroimaging data, a subset of whom had useable blood data (used for quantifying IL-6 and NfL) and completed a cognitive task. Gray matter (GM) thickness values were extracted from brain areas of interest using Freesurfer. Regression models were used to test relationships between IL-6, NfL, GM, and cognitive performance. To test putative functional relationships between these variables, exploratory path analytic models were estimated, in which the relationship between age, IL-6, and working memory performance were linked via four different operationalizations of brain health: (1) a latent GM variable composed of several regions linked to cognitive impairment, (2) NfL alone, (3) NfL combined with the GM latent variable, and (4) the hippocampus alone.

**Results**: Regression models showed that IL-6 and NfL were significantly negatively associated with GM volume and that GM was positively associated with cognitive performance. The path analytic models indicated that age and cognitive performance are linked by GM in the hippocampus as well as several other regions previously associated with cognitive impairment, but not by NfL alone. Peripheral IL-6 was not associated with age in any of the path models.

**Conclusions**: Results suggest that among healthy older adults, there are several GM regions that link age and cognitive performance. Notably, NfL alone is not a sufficient marker of brain changes associated with aging, inflammation, and cognitive performance.

## Introduction

Cognitive functioning is an essential part of everyday living, consisting of a myriad of mental processes used to learn, think, and make decisions. Working memory in particular is a critical executive function involved in many facets of daily life, including concentration, learning new information or skills, reasoning, and decision making (Süß et al., 2002; Baddeley, 2010; Furley and Memmert, 2012). Substantial evidence suggests that cognitive function, and specifically working memory, declines over the course of normal aging (Craik and Salthouse, 2011).

Neurodegenerative diseases are also associated with age such that older individuals are most at risk (Emard et al., 1995). One of the most prevalent neurodegenerative diseases is Alzheimer’s disease (AD), which accounts for 60–80% of dementia cases and affects 10% of the United States population (Zhao et al., 2020). AD is characterized by a decline in memory, as well as language problems. Given the number of individuals affected by age-related cognitive decline and AD, as well as the significant negative impact of AD on daily functioning and quality of life in older adults, investigating the causes and risk factors for age-related cognitive decline and AD is an important area of research. In particular, exploring biological variables that may serve as indicators of the development and progression of cognitive decline is currently a high-priority research area.

One biological factor of particular importance to age-related cognitive decline is altered immune function. Over the course of normal aging, individuals experience an increase in chronic, low-level inflammation throughout the brain and body, which likely contributes to a number of age-related illnesses (Fulop et al., 2018; Aiello et al., 2019). Notably, increases in biological indicators of inflammation in the brain and body have been linked to cognitive impairment, and this appears to be particularly true in older adults. For example, studies have found associations between inflammation and cognitive functioning in older adults (Tangestani Fard and Stough, 2019). Studies also show that inflammation is associated with reduced gray matter (GM) volume (Marsland et al., 2008; Satizabal et al., 2012; Zhang et al., 2016). Interestingly, inflammatory markers are also associated with increased neurofilament light (NfL) levels (note that NfL is a marker of neuronal injury, with greater NfL levels suggesting more neuronal injury; Hsiao et al., 2020). The latter relationship between inflammation and brain changes (GM volume reduction and increased NFL levels) is especially interesting given the additional associations between GM and AD (Thompson et al., 2003; Karas et al., 2004; Busatto et al., 2008), GM and cognition (Zimmerman et al., 2006; Kramer et al., 2007) and NFL and AD (Petzold et al., 2007; Olsson et al., 2016). Taken together, there is a wealth of data connecting inflammation, brain changes, cognitive decline, and AD.

While it is critical that researchers continue investigating the determinants and markers of AD and other dementias, it is also important to examine how these variables may relate to cognitive decline within healthy, aging populations. Working memory functions are necessary for completing all manner of everyday activities, yet healthy older adults show reduced working memory capacity (the amount of information that can be held in working memory at one time) and differential brain activation during working memory tasks compared to younger adults (Kirova et al., 2015), making even nonclinical working memory deficits an important area of research. Additionally, exploring biological avenues that may impact cognition and memory in non-clinical populations may inform pre-disease functioning and help with the early diagnosis of AD.

Finally, there is emerging evidence that cannabinoid compounds present in the cannabis plant [e.g., delta-9- tetrahydrocannabinol (THC)] may influence cognitive function across the lifespan (Sagar and Gruber, 2019; Scott et al., 2019). Cannabis acts on the endogenous cannabinoid system (eCS), which appears to have some neuroprotective effects (Paloczi et al., 2018). For example, decreasing inflammation may be one mechanism through which cannabis could exert positive effects (Namdar and Koltai, 2018). In the context of increasing cannabis legalization in the United States and elsewhere, and cannabis use is becoming increasingly common among older adults (Han and Palamar, 2020). Notably, however, eCS signaling declines with age (Di Marzo et al., 2015), and there is limited research on the neural and health impacts of cannabis use among older adults. It is also relevant to note that chronically-administered THC may reverse age-related cognitive impairment in animal models (Bilkei-Gorzo et al., 2017). Thus, it is important to consider the role that cannabis use might play in the relationships between aging, inflammation, and cognitive function in humans.

The present study aimed to examine the relationship between age, peripheral inflammation, brain health, and working memory in a sample of healthy older adults (ages 60–88; *N* = 273). Analyses were conducted on a smaller subset of participants from this study who had useable MRI scan data (*n* = 173). Specifically, we ran a series of regression models looking at the relationships between age, IL-6 and NfL measured in peripheral blood, GM volume assessed via magnetic resonance imaging, and performance on a working memory task. Additionally, to test hypothesized functional relationships between these variables, we ran four exploratory path analytic models in which the relationship between age, IL-6, and working memory performance were linked via different pathways associated with brain health (e.g., age predicting IL-6; IL-6 predicting NfL/GM volume, and NfL/GM volume predicting working memory performance). It was hypothesized that age would be positively associated with IL-6, IL-6 would be negatively associated with GM volume and positively associated with NfL, and GM volume would be positively associated with working memory while NfL would be negatively associated with working memory. Given that 15 subjects in the present study reported using cannabis at least weekly, we controlled for the possible relationship between current cannabis use and markers of brain health in the exploratory path models and also conducted exploratory comparisons of GM volume between the cannabis users and non-users in the sample.

## Materials and Methods

### Procedures

#### Study Sample and Recruitment

This study was part of a larger project investigating the effect of exercise (i.e., moderately intense continuous training with interval training vs. low intensity continuous training) on social, emotional, and cognitive functioning among older adults (i.e., 60 + years old). The current analysis included baseline data collected prior to participant randomization to an exercise intervention among a sample of older adults who completed neuroimaging scans, blood work, and/or cognitive tasks. Note that *N* = 273 total older adults took part in the study, and a subset of these (*n* = 173) completed the MRI scanning session. A subset of these individuals also had useable blood data (used for quantifying IL-6 and NfL) and completed a cognitive task (see “Participants Characteristics” section below for sample size information for each analysis). Recruitment and data collection took place in the Denver-Boulder, Colorado area between April 2014 and November 2018. Multiple recruitment strategies were used including Craigslist, ResearchMatch, targeted mailings, electronic advertisements placed in campus bulletins, flyers posted on campus and at community centers and recreation centers, and advertisements on social media websites. Participants were eligible for inclusion if they were aged 60 or over, if they reported less than 80 min per week of moderate intensity physical activity in the last 6 months as reported at screening, they were physically capable of safely engaging in moderate intensity exercise as assessed by the study physician, able to successfully complete a maximal oxygen uptake (VO_2_max) test without evidence of cardiac or other abnormalities, and able to make fewer than three errors on the Pfeiffer Mental Status test (Pfeiffer, 1975). The weekly fitness threshold for inclusion was selected because the goal of the larger study was to work with a population that was not already highly active.

Participants were excluded if they reported heavy smoking (>20 pack years), uncontrolled diabetes (hemoglobin A1C >7%) or diabetes treated by insulin or sulfonylureas, uncontrolled hypertension (systolic blood pressure > = 160 mmHg or diastolic blood pressure > = 100 mmHg), magnetic resonance imaging (MRI) contraindications or body size exceeding the capacity of the MRI (given that aspects of the larger study involved structural and functional MRI measures), current use of antipsychotic medications, or diagnosis of bipolar disorder, schizophrenia, dementia, or Alzheimer’s disease. Study procedures were reviewed and approved by the University’s Institutional Review Board.

#### Study Design

The data presented here were collected as part of a larger randomized controlled trial with multiple aims called Fitness, Older Adults, and Resting State Connectivity Enhancement (FORCE). Funding for this study was provided by R01AG43452 (NIA; Clinical-trials.gov, identifier: NCT0206861). The overall aim of FORCE was to examine the relationship between changes in physical activity and changes in brain network connectivity and cognitive, social, emotional, and financial functioning among older adults. However, the present analysis is only focused on the baseline data, thus the exercise sessions and follow-up visits are not discussed. Additional information about the FORCE methods has been published previously (Martin-Willett et al., 2021, 2020).

#### MRI Acquisition

Scan data were acquired on a Siemens 3T MRI scanner with a 32-channel head coil at the Intermountain Neuroimaging Consortium at the University of Colorado Boulder. Scan data acquired prior to April 2016 were collected on a TRIO system. In April/May 2016 the TRIO system was upgraded to a Prisma Fit system; data acquired after May 2016 were collected on this system.

Each participant underwent a multi-echo MPRAGE (magnetization prepared rapid acquisition with gradient echo) T1 weighted anatomical scan (TR = 2,530 ms, TE = 1.64 ms, flip angle = 7°, FOV = 256 mm × 256 mm). A field map was also acquired to reduce RF inhomogeneities and spatial distortion (TR = 400 ms, TE = 4.92 ms, FOV = 238 mm × 238 mm).

### Measures

#### Demographics

Participants filled out questionnaires on demographic information including their gender, age, race, marital status, income, and education level.

#### Working Memory Assessment

Cognitive functioning was measured via the Keep Track task (Yntema and Mueser, 1962; Yntema, 1963). In this task, a series of words belonging to one of six categories (relatives, distances, metals, animals, colors, and countries) was presented one at a time. After two practice trials, participants completed nine trials in which they were asked to keep track of and report the last word presented from two to four categories. Three trials included two categories, three trials included three categories, and three trials included four categories. Each word was presented for 2 s, with the applicable categories at bottom of the screen. The outcome that was analyzed in the present study was the number of correct words remembered.

#### Blood Samples

Ten ml of whole blood was drawn to measure protein levels of the proinflammatory cytokine IL-6, as well as protein levels of circulating NfL. The cytokine was assayed using the BioLegend LEGENDplexTM bead-based immunoassay and flow cytometry procedures (BioLegend, San Diego, CA, USA). Following procedures from prior cytokine studies (Ostrowski et al., 1999), the log of IL-6 values was calculated for use in all analyses, due to non-normal distribution of data. We also measured Neurofilament-Light (NfL) protein levels in the blood plasma samples using the UmanDiagnostics NF-Light assay (Quanterix Corp, Billerica MA, USA).

### Gray Matter ROI Selection

GM analyses involved the creation of an “Alzheimer’s Disease Cortical Thickness Signature” involving the averaged Freesurfer thickness values across eight bilateral brain regions (inferior temporal, middle temporal, inferior parietal, fusiform, precuneus, superior parietal, temporal pole, and entorhinal regions), following prior research indicating that this signature is indicative of memory function (Busovaca et al., 2016; Allison et al., 2019). We also tested associations between predictors of interest and the average of the left and right hippocampus volume, given consistent data linking aging, decreased hippocampal volume, and cognitive decline (Bettio et al., 2017).

### Data Analysis

#### Structural Analysis

Images were converted from DICOM to NIFTI and visually inspected for gross artifacts. Following visual QA checks, automatic cortical and subcortical segmentation and parcellation were performed in Freesurfer’s automated Recon-all pipeline (7.1.0^1^). Intensities attributed to magnetic inhomogeneities were corrected and normalized, images were then skull-stripped and segmented into gray/white matter and cerebrospinal fluid following image intensities and gradients. Triangular tessellation was then applied to the resulting image to provide a smooth representation of the gray/white matter interaction. For more technical details see Dale et al. (1999) and Fischl et al. (1999, 2002). Cortical thickness values were exported from Freesurfer for use in all analyses.

### Statistical Analysis Plan

Pearson correlations were run between age and all outcomes of interest (GM, NfL, IL-6, and performance on the Keep Track task). Next, for each relationship of interest, we conducted Ordinary Least Squares (OLS) regression models in SPSS (Version 27, IBM), in which each outcome variable of interest was regressed on the predictor of interest. In all models involving GM, the total estimated intracranial volume was included as a covariate. For models in which GM was the criterion, one regression was conducted using the average of all voxels in the AD signature ROI as the outcome variable in order to decrease Type I error. As a sensitivity analysis, additional models were then tested including gender and cannabis use in all regressions in which a significant association emerged between the outcome variable and the predictor of interest. In all regression models reported below, slope values are reported as standardized regression coefficients. The significance level was set at *p* < 0.05 for all outcomes.

**Figure 1:**
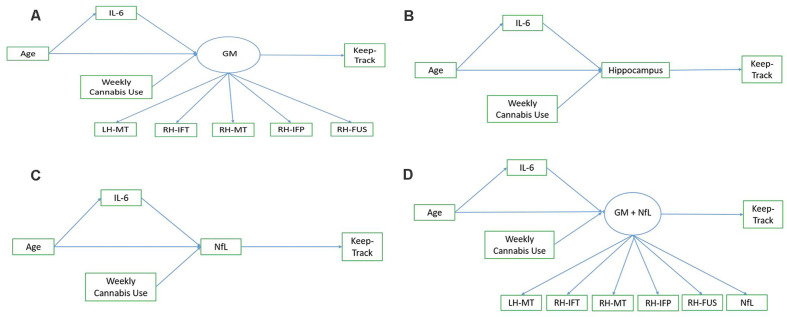
Conceptual models. Panel **(A)** shows the conceptual model of the relationship between age, IL-6, the frontal gray matter (GM) latent variable, and Keep Track. Panel **(B)** shows the conceptual model of the relationship between age, IL-6, hippocampal volume, and Keep Track. Panel **(C)** shows the conceptual model of the relationship between age, IL-6, NfL, and Keep Track. Panel **(D)** shows the conceptual model of the relationship between age, IL-6, frontal GM/NfL latent variable, and Keep Track. Note. LH-MT, left hemisphere middle temporal thickness; RH-IFT, right hemisphere inferior temporal thickness; RH-MT, right hemisphere middle temporal thickness; RH-IFP, right hemisphere inferior parietal thickness; RH-FUS, right hemisphere fusiform thickness; NfL, Neurofilament light polypeptide.

Exploratory path analysis of the relationship between age, IL-6, Keep Track performance, and “brain health” variables as assessed by either (1) a latent frontal GM variable, (2) average right and left hippocampal volume, (3) NfL, or (4) a latent variable combination of frontal GM and NfL, were run in R using the sem package (Fox et al., 2020). The latent frontal GM variable was empirically created by running correlations between each of the 16 regions (eight bilateral regions) included in the AD signature and then selecting the five regions that were correlated with at least three of the variables of interest (age, Keep Track, NfL, and IL-6). We investigated four models, with a different brain health measure used in each model. In all models, age was included as a predictor of IL-6 and the brain health measure of interest (either the latent GM variable, hippocampal volume, NfL, or latent GM+NfL), IL-6 was a predictor of brain health, and brain health was a predictor of Keep Track performance. Additionally, all models included weekly cannabis use (1 = used weekly vs. 0 = did not use weekly) as a predictor of brain health, given that the sample included a small number (*n* = 15) of weekly cannabis users. The latent frontal GM variable was comprised of the following five gray matter density variables: left hemisphere middle temporal thickness, right hemisphere inferior temporal thickness, right hemisphere middle temporal thickness, right hemisphere inferior parietal thickness, and right fusiform thickness (and NfL, in the last model). See Figure 1 for conceptual models of all path analyses.

For the models in which a latent variable was used, measurement models of the latent variables were first conducted. For both measurement models and path analytic models, the fit was assessed via the χ^2^ likelihood ratio test, Root Mean Square Error Of Approximation (RMSEA), the 95% confidence interval of the RMSEA (95%CIs), Standardized Root Mean Square Residual (SRMR), and the Comparative Fit Index (CFI). Because the models are not nested and, thus, not directly comparable via *χ*^2^ difference tests, we also include the Akaike’s Information Criterion (AIC) to compare the relative fit of non-nested models.

Finally, as an exploratory analysis, we compared the cannabis users and non-cannabis users in the sample on GM volumes in the hippocampus and several other subcortical structures.

## Results

### Participant Characteristics

In total, *N* = 173 individuals had useable GM scan data. As can be seen in Table 1, participants were mostly female and reported minimal depression and anxiety. Note that of the 173 participants with scan data, 112 had useable IL-6 data, 167 had useable Keep Track data and 115 had useable NfL data. Thus, the sample size differs depending on the variables included in the analysis.

**Table 1:** Sample demographics.

Characteristic	Total scanned participants (*N* = 173) % or Mean (*SD*)
Age	67.10 (5.3)
Gender (% female)	63%
Ethnicity	
Unknown/not reported	1.7%
Hispanic or Latino	3.5%
Not-Hispanic or Latino	94.8%
Race	
Unknown/not reported	1.7%
More than one race	1.2%
Asian	4.6%
White	92.5%
Highest Level of Education^a^
Less than high school	0.6%
High School or GED	1.7%
Some College	9.8%
Associates Degree or Technical Certification	4.6%
Bachelors Degree	37.6%
Masters Degree	28.9%
Doctoral Degree	15.0%
Employment^b^	
Homemaker or stay at home parent	4.0%
Unemployed/unable to work/retired/other	46.2%
Part time (work less than 30 h/week)	21.4%
Full time (work more than 30 h/week)	26.0%
Baseline Beck Depression Inventory Total^c^	7.68 (6.3)
Baseline Beck Anxiety Inventory Total^c^	4.78 (4.7)

*Note. ^a^Three participants were missing data on education level; ^b^four participants were missing data on employment; ^c^one participant was missing the Beck Depression Inventory (BDI) scale and Beck Anxiety Inventory (BAI) scale*.

### Correlations Between Age and Keep Track Performance, IL-6, NfL, and GM

Age was positively correlated with IL-6 (*r* = 0.237, *p* = 0.012 *n* = 112), positively correlated with NfL (*r* = 0.519, *p* = < 0.001, *n* = 130) and negatively correlated with the number of correct responses on the Keep Track task (*r* = −0.265, *p* < 0.001, *n* = 193). There was also a significant partial correlation between age and AD signature, covarying for total estimated intracranial volume (partial *r* = −0.312, *p* < 0.001, *n* = 173) and a significant partial correlation between age and hippocampus volume, covarying for total estimated intracranial volume (partial *r* = −0.510, *p* < 0.001, *n* = 170). Results of partial correlations (covarying for ICV) between age and the individual regions within the AD signature are included in Table 2.

**Table 2:** Correlations between individual regions within AD signature and variables of interest.

Region within AD signature	IL-6 (*n* = 112)	NfL (*n* = 115)	Keep Track (# individual responses correct; *n* = 167)	Age (*n* = 173)
lh_entorhinal_thickness	*r* = −0.191, *p* = 0.044	*r* = 0.003, *p* = 0.975	*r* = 0.124, *p* = 0.112	*r* = −0.085, *p* = 0.265
lh_inferiortemporal_thickness	*r* = −0.010„ *p* = 0.918	*r* = −0.377, *p* < 0.001	*r* = 0.115, *p* = 0.140	*r* = −0.169, *p* = 0.027
lh_middletemporal_thickness	*r* = −0.241, *p* = 0.011	*r* = −0.320, *p* < 0.001	*r* = 0.084, *p* = 0.282	*r* = −0.343, *p* < 0.001
lh_inferiorparietal_thickness	*r* = −0.159, *p* = 0.097	*r* = −0.140, *p* = 0.137	*r* = 0.101, *p* = 0.197	*r* = −0.205, *p* = 0.007
lh_fusiform_thickness	*r* = 0.000, *p* = 0.998	*r* = −0.225, *p* = 0.016	*r* = 0.119, *p* = 0.127	*r* = −0.270, *p* < 0.001
lh_precuneus_thickness	*r* = −0.055, *p* = 0.563	*r* = −0.177, *p* = 0.060	*r* = 0.075, *p* = 0.335	*r* = −0.224, *p* = 0.003
lh_superiorparietal_thickness	*r* = −0.125, *p* = 0.190	*r* = −0.096, *p* = 0.307	*r* = 0.067, *p* = 0.393	*r* = −0.170, *p* = 0.026
lh_temporalpole_thickness	*r* = −0.134, *p* = 0.161	*r* = −0.117, *p* = 0.217	*r* = 0.173, *p* = 0.026	*r* = −0.017, *p* = 0.821
rh_entorhinal_thickness	*r* = −0.125, *p* = 0.190	*r* = −0.117, *p* = 0.213	*r* = 0.115, *p* = 0.140	*r* = −0.111, *p* = 0.149
rh_inferiortemporal_thickness	*r* = −0.199, *p* = 0.036	*r* = −0.234, *p* = 0.012	*r* = 0.249, *p* = 0.001	*r* = −0.365, *p* < 0.001
rh_middletemporal_thickness	*r* = −0.201, *p* = 0.034	*r* = −0.331, *p* < 0.001	*r* = 0.213, *p* = 0.006	*r* = −0.421, *p* < 0.001
rh_inferiorparietal_thickness	*r* = −0.075, *p* = 0.435	*r* = −0.188, *p* = 0.045	*r* = 0.179, *p* = 0.021	*r* = −0.337, *p* < 0.001
rh_fusiform_thickness	*r* = −0.161, *p* = 0.092	*r* = −0.251, *p* = 0.007	*r* = 0.185, *p* = 0.017	*r* = −0.318, *p* < 0.001
rh_precuneus_thickness	*r* = −0.224, *p* = 0.018	*r* = −0.141, *p* = 0.136	*r* = 0.116, *p* = 0.137	*r* = −0.326, *p* < 0.001
rh_superiorparietal_thickness	*r* = −0.101, *p* = 0.294	*r* = −0.081, *p* = 0.394	*r* = 0.053, *p* = 0.495	*r* = −0.233, *p* = 0.002
rh_temporalpole_thickness	*r* = 0.156, *p* = 0.102	*r* = −0.258, *p* = 0.006	*r* = 0.102, *p* = 0.192	*r* = −0.112, *p* = 0.142

### Associations Between NfL and GM and Between IL-6 and GM

**Figure 2:**
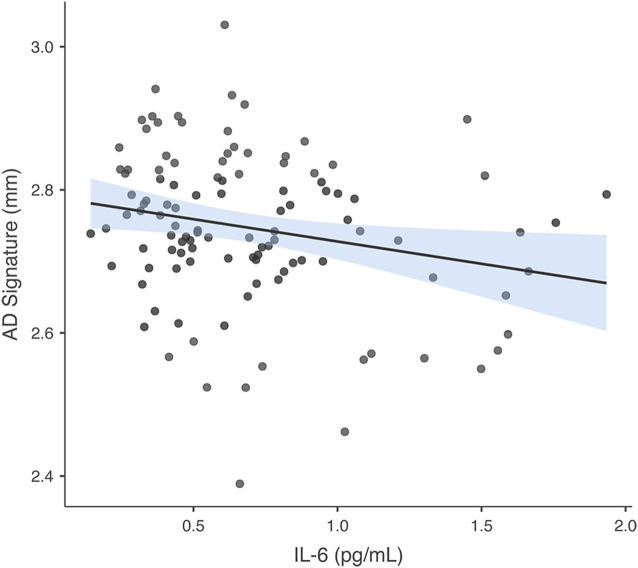
Association between AD signature and IL-6. Scatterplot depicting the raw association between the AD Signature mean cortical thickness and IL-6.

In the regression model in which GM thickness within the AD signature was the criterion and IL-6 was the predictor of interest (covarying for ICV, as noted in the *Analysis Plan* for all GM models), predictors accounted for 6.3% of the variance in GM. Inspection of individual regression slopes indicates that IL-6 was significantly negatively associated with GM thickness within the AD signature *b* = −0.225, *t*_(109)_ = −2.429, *p* = 0.017. See Figure 2 for a scatterplot showing the raw association between the AD signature and IL-6. In the model in which hippocampus volume is the criterion and IL-6 the predictor of interest, predictors accounted for 9.5% of the variance in hippocampus volume. Inspection of individual regression slopes indicates that IL-6 was significantly negatively associated with hippocampus volume *b* = −0.273, *t*_(109)_ = −2.598, *p* = 0.011.

**Figure 3:**
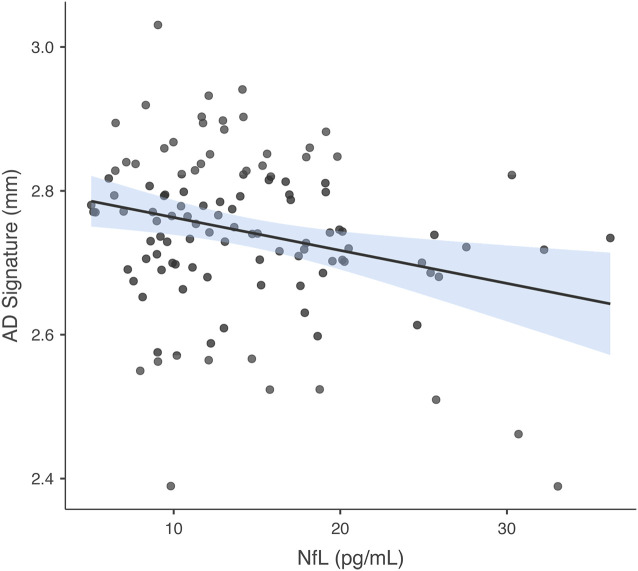
Association between AD Signature and NfL. Scatterplot depicting the raw association between the AD Signature mean cortical thickness and NfL.

In the model in which GM thickness within the AD signature was the criterion and NfL was the predictor of interest, predictors accounted for 8.6% of the variance in GM. NfL was significantly negatively associated with GM thickness within the AD signature *b* = −0.279, *t*_(112)_ = −3.065, *p* = 0.003. See Figure 3 for a scatterplot showing the raw association between the AD signature and NfL. In the model in which hippocampus volume was the criterion and NfL was the predictor of interest, predictors accounted for 13.3% of the variance in hippocampus volume. NfL was significantly negatively associated with hippocampus volume, *b* = −0.334, *t*_(112)_ = −3.765, *p* < 0.001. The partial correlations between IL-6 and NfL and the individual brain regions within the AD Signature are listed in Table 2.

### Associations Between Cognitive Performance and NfL, IL-6, and GM

**Figure 4:**
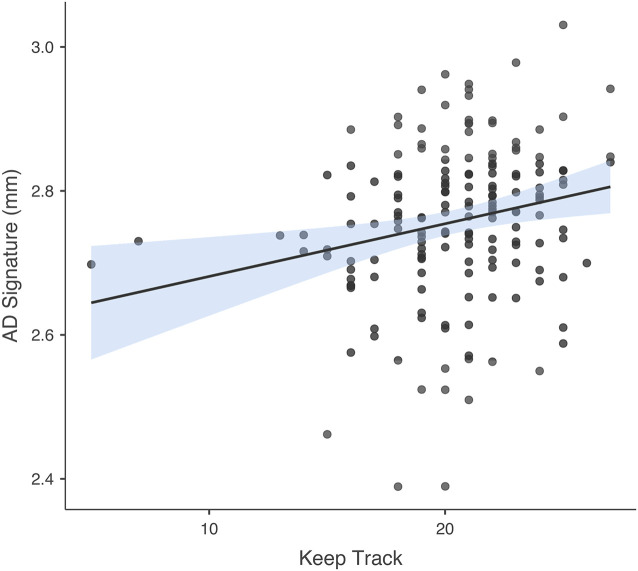
Association between AD signature and Keep Track task. Scatterplot depicting the raw association between the AD Signature mean cortical thickness and the Keep Track task.

The number of individual responses correct on the Keep Track task was not significantly associated with IL-6 or NfL in either regression model. In the model in which AD signature was the criterion and Keep Track task performance was the predictor of interest, predictors accounted for 6.6% of the variance in GM. Keep track performance was significantly positively associated with GM thickness within the AD signature *b* = 0.209, *t*_(164)_ = 2.766, *p* = 0.006. See Figure 4 for a scatterplot showing the raw association between AD signature and Keep Track performance. The partial correlations between Keep Track and the individual brain regions within the AD Signature are listed in Table 2.

**Figure 5:**
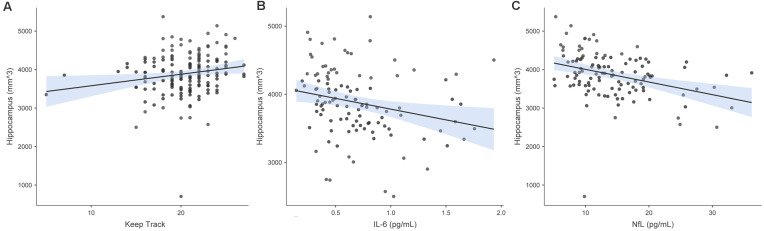
Associations between hippocampal volume and Keep Track, IL-6, and NfL. Panel **(A)** shows the raw association between bilateral hippocampus volume and Keep Track. Panel **(B)** shows the raw association between bilateral hippocampus volume and IL-6. Panel **(C)** shows the raw association between bilateral hippocampus volume and NfL.

In the model in which hippocampus volume was the criterion and Keep Track performance was the predictor of interest, predictors accounted for 5.8% of the variance in hippocampus volume. Keep Track performance was significantly positively associated with the hippocampus volume *b* = 0.198, *t*_(164)_ = 2.538 *p* = 0.012. Figure 5 shows the raw associations between hippocampus volume and Keep Track, IL-6 and NfL.

### Sensitivity Analyses

For each of the significant associations in regression models reported above, we repeated analyses including gender. When gender was included in the models, all of the associations reported above remained significant. We also included cannabis user status in all models, and all associations remained significant.

### Path Analyses

**Figure 6:**
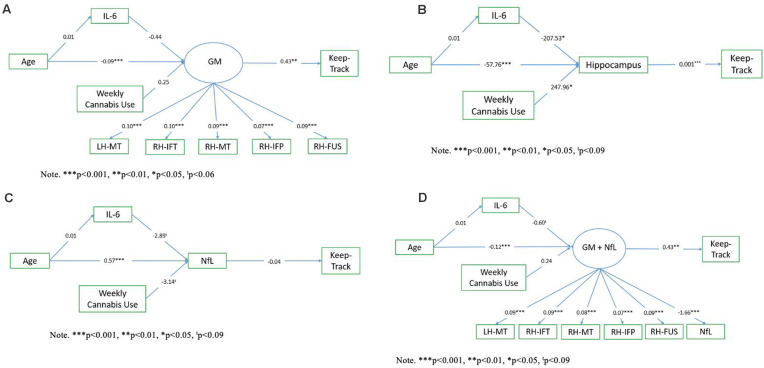
Path analysis results. Panel **(A)** shows the latent path analysis of the relationship between age, IL-6, the frontal GM latent variable, and Keep Track. Panel **(B)** shows the latent path analysis of the relationship between age, IL-6, hippocampal volume, and Keep Track. Panel **(C)** shows the path analysis of the relationship between age, IL-6, Nfl, and Keep Track. Panel **(D)** shows the latent path analysis of the relationship between age, IL-6, frontal GM, and NfL latent variable, and Keep Track. Note. LH-MT, left hemisphere middle temporal thickness; RH-IFT, right hemisphere inferior temporal thickness; RH-MT, right hemisphere middle temporal thickness; RH-IFP, right hemisphere inferior parietal thickness; RH-FUS, right hemisphere fusiform thickness.

The measurement models for both the frontal GM latent variable and the frontal GM latent variable with NfL demonstrated good fit to the data; $\chi_{\left(9\right)}^{2}$ = 6.96, *p* = 0.64, CFI = 1.00, RMSEA = 0.00 (95%CI = [0.00 0.09]), SRMR = 0.03 and $\chi_{\left(5\right)}^{2}$ = 4.28, *p* = 0.51, CFI = 1.00, RMSEA = 0.00 (95%CI = [0.00 0.10]), SRMR = 0.02, respectively. In both cases, all indicators significantly loaded onto the factor (*ps* < 0.001). Model fits for the structural path analyses of models 1, 2, and 4 (using the frontal GM latent variable, hippocampal volume, and the frontal GM/NfL latent variable, respectively) were acceptable while model fit for model 3 (using just NfL) was relatively poor (see Table 3). Within the three well-fitting models, age was not significantly associated with IL-6 but was significantly associated with the brain health variables, which were significantly associated with Keep Track performance (see Figure 6). Additionally, the paths from IL-6 to frontal GM with and without NfL were non-significant, while the path from IL-6 to the hippocampal volume was significant (see Figure 6). Thus, increases in age were significantly, negatively associated with frontal GM with and without NfL and hippocampal volume but not IL-6. IL-6 was in turn not significantly associated with the brain variables except for hippocampal volume, and all brain variables were significantly, positively associated with working memory performance.

**Table 3:** Model fit indices of the path analytic models.

Model fit indices	Model 1	Model 2	Model 3	Model 4
AIC value	−353.90	1849.60	997.39	250.77
CFI	0.97	0.97	0.72	0.91
RMSEA	0.05	0.09	0.15	0.09
95% CI RMSEA	0.00–0.10	0.00–0.19	0.06–0.25	0.05–0.13
SRMR	0.06	0.06	0.09	0.07

*Note. Model 1, model using frontal GM latent variable; model 2, model using hippocampal volume; model 3, model using NfL; model 4, model using frontal GM and NfL latent variable*.

### Cannabis Use Group Differences

**Figure 7:**
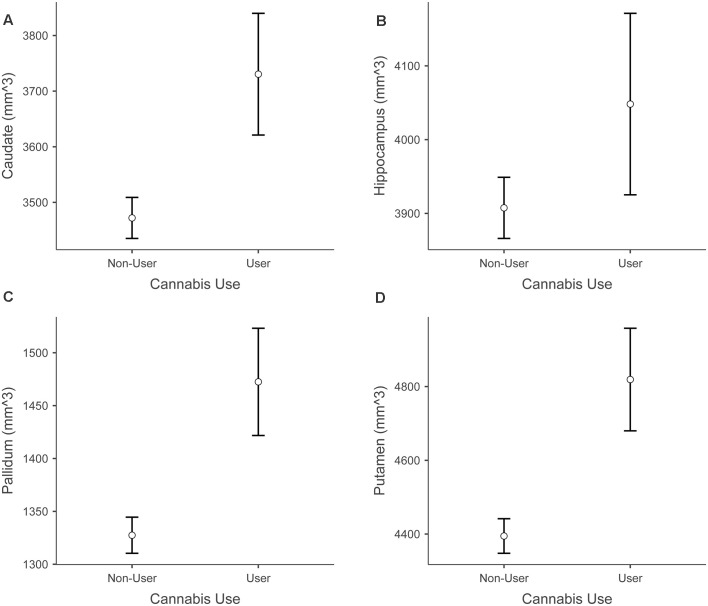
Differences between cannabis users and non-users across subcortical volumes. Panel **(A)** shows increased caudate volume in cannabis users vs. non-users. Panel **(B)** shows increased hippocampus volume in cannabis users vs. non-users. Panel **(C)** shows increased pallidum volume in cannabis users vs. non-users. Panel **(D)** shows increased putamen volume in cannabis users vs. non-users. Error bars depict standard error.

Because path analyses demonstrated that cannabis use status [individuals who use cannabis at least weekly (*n* = 15) vs. individuals who do not use cannabis or use less often than weekly (*n* = 132)] was a significant predictor of hippocampal volume, we used *post hoc* independent samples *t*-tests to compare the mean GM volumes of the hippocampus and several other subcortical structures (bilateral putamen, pallidum, thalamus, caudate). Results of these exploratory *t*-tests demonstrated that the cannabis use group had significantly higher volumes than the non-use group in the putamen, *t*_(145)_ = −2.890, *p* = 0.004, Cannabis Use Group Mean = 4819.14 (524.1), Non-Use Group Mean = 4394.67 (540.5), pallidum *t*_(145)_ = −2.711, *p* = 0.008, Cannabis Use Group Mean = 1472.48 (235.7), Non-Use Group Mean = 1327.47 (191.7) and caudate *t*_(145)_ = −2.240, *p* = 0.027, Cannabis Use Group Mean = 3730.48 (425.8), Non-Use Group Mean = 3472.06 (423.2). No significant group differences emerged for the other regions tested. Figure 7 shows cannabis use group differences in these subcortical structures.

## Discussion

The present study explored relationships between aging, circulating IL-6 in blood samples, GM, NfL from peripheral blood, and cognitive performance using the Keep Track Task. We observed the expected associations between age and all of these variables, such that age was associated with higher levels of IL-6 and NfL in the blood and with lower GM in the AD signature ROI and hippocampus, as well as worse performance on the Keep Track task. Regression models also supported overall hypotheses, indicating that IL-6 and NfL were both significantly negatively associated with GM in the AD signature and hippocampus, consistent with the idea that NfL is a biomarker indicative of neuronal damage (Hsiao et al., 2020). Further, we observed expected positive associations between GM variables and Keep Track performance, consistent with data suggesting that decreases in GM are associated with a decline in cognitive function in normal aging (Zimmerman et al., 2006; Kramer et al., 2007).

Conversely, no associations were observed between Keep Track performance and IL-6 or NfL. This suggests that although IL-6 and NfL may be useful biomarkers related to neuronal damage, they may be too distal from the cognitive performance phenotype to show meaningful associations. We also conducted an exploratory comparison of four different path analytic models in order to further examine putative mechanistic relationships between IL-6, GM volumes, NfL, and Keep Track performance. Each model included a different operationalization of brain health. The models were as follows: Model 1 (a latent GM variable comprised of five regions from the AD signature that were chosen because they were correlated with at least three of variables of interest, see Table 2), Model 2 (hippocampus volume), Model 3 (NfL), and Model 4 (a latent variable comprised of NfL and the latent GM variable). Model 3 showed a poor fit to the data, suggesting that NfL alone is not a sufficient marker of brain changes associated with aging, inflammation, and cognitive performance. This model was tested due to the suggestion that NfL could potentially be a useful biomarker of neural damage (Alirezaei et al., 2020), particularly in certain clinical populations such as individuals with neurological disorders (Gaetani et al., 2019), and if this were the case, the use of NfL as a clinical blood-derived biomarker would have significantly fewer logistical and financial barriers than MRI scans and may be more accessible for both research and clinical purposes. However, these data suggest that, at least in the context of healthy aging, NfL is not an adequate predictor of cognitive performance and is not associated with inflammation.

The other three models all demonstrated adequate fit to the data. Recall that in *bivariate* correlations age was strongly related to IL-6 (Singh and Newman, 2011). However, there was no significant association between age and IL-6 in the context of the three adequately-fitting *multivariate* path models in which both IL-6 and age were predictors of brain health. This supports the idea that inflammation may be a stronger proximal determinant of brain health than simple chronological age.

In all three adequately-fitting path analytic models, age was associated with the brain health variable, which was significantly related to cognitive performance. Notably, only the model in which the hippocampus volume was the brain health variable of interest showed a significant association between IL-6 and brain health. This is consistent with existing studies suggesting that the hippocampus may be particularly impacted by inflammation in normal aging and in age-related diseases. For example, in middle-aged adults, IL-6 is negatively related to GM volume in the hippocampus (Marsland et al., 2008). There is also a relationship between IL-6 and the hippocampus in AD patients. Specifically, one of the hallmarks of AD pathology is the accumulation of beta-amyloid in the hippocampus (Lazarov et al., 2002). The accumulation of beta-amyloid peptide in the AD brain initiates a neuroinflammatory response which includes the production of IL-6 and other cytokines (Tuppo and Arias, 2005). In an early study, hippocampal and cortical brain tissue from AD patients was found to have increased levels of IL-6 (Strauss et al., 1992). Rodent studies also support this relationship. For example, in a transgenic mouse model of AD, increased IL-6 mRNA in the hippocampus was observed in both younger and older transgenic mice, compared to wild-type mice (Tehranian et al., 2001). Our findings are in line with prior work and underscore the potential importance of inflammation specifically within the hippocampus in mediating cognitive function in both normal aging and age-related diseases.

Finally, we included cannabis as a covariate in all models, given the potential neuroprotective role of the eCS (Paloczi et al., 2018), the potential for cannabis to reverse age-related cognitive decline in animal models (Bilkei-Gorzo et al., 2017), and the fact that older individuals (including 15 individuals in the present study) are increasingly reporting regular cannabis use (Han and Palamar, 2020). Because cannabis use was associated with hippocampal volume in the path model, we conducted exploratory comparisons of GM volume between cannabis users and non-users across several subcortical structures. Group differences emerged in the putamen, pallidum, and caudate, such that the cannabis use group had higher GM volumes in these areas. Because of the small number of regular cannabis users, these findings should be viewed with caution and considered preliminary. However, in our prior work in an aging sample, we also found differences in gray matter density between individuals who use cannabis and those who do not, such that those who use cannabis had greater gray matter volumes in the putamen, lingual cortex, and rostral middle frontal cortex compared to those who do not use cannabis (Thayer et al., 2019).

## Limitations and Future Directions

The current study has several methodological limitations worth noting. First, the cross-sectional nature of the data precludes drawing causal conclusions regarding the observed relationships between aging, IL-6, GM, NfL, and cognitive performance. Future studies should examine these variables in the context of a longitudinal design, which would support conclusions about cause and effect. Another limitation of note is the fact that we had slightly different sample sizes for each analysis, due to the limited availability of scan data and blood data for certain participants. We also had a very small number of cannabis users in the sample, which limits our ability to draw strong conclusions about the possible effects of cannabis use in an older adult population. Finally, it should be noted that the older adults included in this study were generally healthy (i.e., they had no major health conditions and were recruited into the study on the basis of being healthy enough to safely engage in regular exercise), thus these results may not generalize to the broader older adult population. Strengths of the study include the large sample with neuroimaging data and the inclusion of novel biomarkers. Findings from this study suggest that future, large-scale longitudinal studies should explore the relationship between age-related changes in the eCS, cannabis use, and GM volume in older adults. Further exploring these relationships may be of particular interest among older adults with mild cognitive impairment and early AD.

## Data Availability Statement

The raw data supporting the conclusions of this article will be made available by the authors, without undue reservation.

## Ethics Statement

The studies involving human participants were reviewed and approved by University of Colorado IRB. The patients/participants provided their written informed consent to participate in this study.

## Author Contributions

HK and KH developed the idea. AB provided oversight of the study and obtained funding. HK wrote the manuscript and ran analyses. EM ran neuroimaging analyses. CS and AB ran path analyses and assisted with manuscript idea development. All authors contributed to the article and approved the submitted version.

## Conflict of Interest

The authors declare that the research was conducted in the absence of any commercial or financial relationships that could be construed as a potential conflict of interest.

## Publisher’s Note

All claims expressed in this article are solely those of the authors and do not necessarily represent those of their affiliated organizations, or those of the publisher, the editors and the reviewers. Any product that may be evaluated in this article, or claim that may be made by its manufacturer, is not guaranteed or endorsed by the publisher.

## Footnotes

^1^ http://surfer.nmr.mgh.harvard.edu
